# Electrochemical Characterization of Iron (III) Doped Zeolite-Graphite Composite Modified Glassy Carbon Electrode and Its Application for AdsASSWV Determination of Uric Acid in Human Urine

**DOI:** 10.1155/2019/6428072

**Published:** 2019-05-02

**Authors:** Meareg Amare

**Affiliations:** Department of Chemistry, Bahir Dar University, Ethiopia

## Abstract

Iron (III) doped zeolite/graphite composite modified glassy carbon electrode was prepared for determination of uric acid in human urine samples. Electrochemical impedance spectroscopic and cyclic voltammetric results confirmed surface modification of the surface of glassy carbon electrodes. Appearance of oxidative peak current with an over threefold enhancement at significantly reduced overpotential for uric acid at the composite modified electrode relative to the unmodified and even graphite modified electrode confirmed the electrocatalytic property of the composite towards electrochemical oxidation of uric acid. Under optimized method and solution parameters, linear dependence of peak current on uric acid concentration in a wide range of 1-120 *μ*M, low detection limit value (0.06 *μ*M), replicate results with low RSD, and excellent recovery results (96.61-103.45%) validated the developed adsorptive anodic stripping square wave voltammetric (AdsASSWV) method for determination of uric acid even in aqueous human urine samples. Finally, the developed composite modified electrode was used for determination of uric acid content in human urine samples collected from three young male volunteers. While the uric acid level in the urine samples from two of the studied volunteers was within the normal range, of the third was under the normal range.

## 1. Introduction

**Scheme 1:**
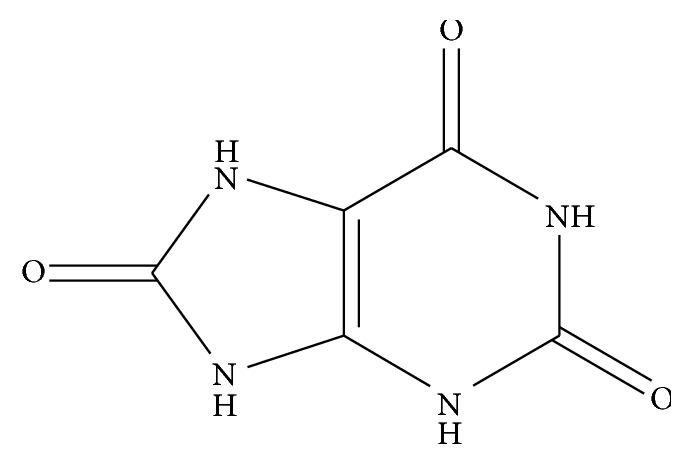
Structural formula of uric acid.

Uric acid (2,6,8-trioxypurine) (Scheme 1) is a compound endogenously produced by animals as a purine metabolite. It is the end product of purine metabolism in humans due to the loss of uricase activity making humans have higher uric acid (UA) level than other mammals [1, 2]. UA is produced by the liver leading to normal uric acid concentration of 3.4–7.2 for men and 2.4–6.1 mg/dL of plasma for women which ultimately is excreted chiefly by the kidneys (65-75%) and intestines (25-35%) [1, 2]. Due to its excellent antioxidant character, UA is the major contributor for the total antioxidant capacity of plasma in human body [3].

As high UA content in body fluids may cause several diseases including gout, kidney diseases, hypertension, and cardiovascular diseases, lower UA level equally provokes diseases like multiple sclerosis, Parkinson's disease, Alzheimer's disease, and optic neuritis [3–6]. Thus, development of a simple, rapid, and sensitive method for continual monitoring of its level in human body fluids including urine is vital. A range of techniques including chromatography [7, 8], electrophoresis [9, 10], potentiometry [11], spectroscopy [12], and amperometry [13] have been reported for determination of UA in different samples.

In spite of all these efforts made, these methods still remained laborious and time-consuming, require sophisticated equipment, and are expensive, which make them unsuitable for rapid detection and restrain their wide applications [14]. Taking into consideration the fact that electrochemical methods are fast, sensitive, less expensive, and environmentally friendly, these methods have received much attention for determination of uric acid regardless of the matrix type. Although several works have been reported on the electrochemical determination of uric acid [15–21], the development of modified electrode which is more stable, easy to prepare, sensitive, and relatively of low cost is still important. While few works on application of metal doped zeolite-modified glassy carbon electrodes have been reported, to the best of our knowledge Fe(III) impregnated zeolite-graphite composite modified glassy carbon electrode (FeZ-G/GCE) for determination of uric acid in human urine has not been reported.

The superior features of zeolite-modified electrodes (ZMEs) for catalysis and analytical application over other modifiers arise from the joined size selectivity and ion-exchange capacity of zeolite molecular sieves [22].

Thus, the aim of this paper was to characterize the FeZ-G/GCE using cyclic voltammetry and electrochemical impedance spectroscopy, investigate the electrochemical behavior of uric acid at the modified electrode, and further evaluate the applicability of the developed adsorptive anodic stripping square wave voltammetric (AdsASSWV) method for determination of uric acid in urine even in the presence of ascorbic acid.

## 2. Experimental

### 2.1. Chemicals and Reagents

Chemicals and reagents are sodium Y zeolite powder (>90% particle size, Merck), anhydrous ferric chloride (99.99%, Merck), graphite powder (Blulux Laboratories (p) Ltd.), polystyrene (Merck), dichloromethane (99.9%, CARLO ERBA reagents), tetrahydrofuran (99.5%, Blulux laboratories (p) Ltd), paracetamol (Sigma-Aldrich, Germany), uric acid (99.0%, Labort Fine Chem Pvt Ltd), ascorbic acid (99.0%, Merck), orthophosphoric acid (85.74%, Fisher Scientific), potassium dihydrogen orthophosphate (99.0%, Titan Biotech Ltd), dipotassium hydrogen orthophosphate (99.0%, Titan Biotech Ltd), and potassium nitrate (>99.0%, Merck). All chemicals and reagents were of analytical grade and hence were used without prior purification.

### 2.2. Apparatus and Instruments

CHI 760d Electrochemical Workstation (Austin, Texas, USA), ultrasonicator (Indiamart), pH meter (Adwa instruments kit), balance (Nimbus ThermoFisher Scientific), and Eppendorf tube were among the instruments and apparatus used.

### 2.3. Electrochemical Measurements

In both the voltammetric and electrochemical impedance spectroscopic analyses, a conventional three-electrode system was employed with a bare GCE (3 mm in diameter), graphite modified glassy carbon electrode (GGCE), or iron (III) doped zeolite/graphite composite modified glassy carbon electrode (FeZGC/GCE) as working electrode, silver/silver chloride (Ag/AgCl) as reference electrode, and platinum coil as a counterelectrode.

While cyclic voltammetry was used to investigate the electrochemical behavior of uric acid at the unmodified and modified glassy carbon electrodes, effect of scan rate on oxidative peak current and dependence of both the oxidative peak current and peak potential of UA at the surface of the composite modified glassy carbon electrode on pH; square wave voltammetry under default square wave parameters (amplitude 25 mV, step potential 4 mV, and frequency 25 Hz); and optimized deposition parameters (E_acc_ of -0.1 V and t_acc_ 30 s) were used for the determination of UA in human urine samples collected from three volunteers. The electrochemical impedance spectroscopic behavior of the assessed working electrodes was also investigated using 10 mM Fe(CN)_6_^3−^/^4−^ in 0.1 M KCl as a probe.

### 2.4. Solution and Sample Preparation

#### 2.4.1. Standard Solution Preparation

100 ml of 10 mM stock solution of UA in pH 7.0 of 0.1 M phosphate buffer solution (PBS) was prepared from which 1 mM working solutions of UA in PBS of various pHs were prepared. Standard working solutions of UA in pH 7.0 PBS were also prepared by serial dilution from the stock solution for calibration curve.

#### 2.4.2. Human Urine Sample Preparation

Three human urine samples designated as “A,” “B,” and “C” were collected from three young male volunteers using cleaned plastic bottles. After the samples were centrifuged for 10 min at 4000 rpm, 50 mL of urine solution was prepared for each urine sample by putting 1 mL of the centrifuged urine in a volumetric flask and diluted to the mark with pH 7.0. The diluted urine solutions were kept in a refrigerator until analysis.

### 2.5. Electrode Preparation

Glassy carbon electrode of 3 mm disc diameter was made ready for use as bare or modified after being polished successively with alumina powder of 1.0, 0.3, and 0.05 *μ*m course size.

A mixture of 50 mg of graphite powder and 5 mg of polystyrene as a binder was dispersed in 250 *μ*L solution of tetrahydrofuran and dichloromethane (2:3 volume ratio). A 2 mL Eppendorf tube containing the mixture was first hand-shaken for five minutes and then sonicated for two minutes in ultrasonic water bath. The graphite modified glassy carbon electrode (G/GCE) was prepared by casting 10 *μ*L of the suspension on the surface of a mirror-like cleaned glassy carbon electrode and air-dried before use.

The iron (III) doped zeolite-graphite composite modified glassy carbon electrode (FeZ-G/GCE) was prepared following reported procedure with minor modification [21]. Briefly, lightly ground 1 g of zeolite y using a mortar and pastel was added to 250 mL of 0.01 M FeCl_3_ in deionized water and stirred with a magnetic stirrer at 2000 rpm for 48 hrs. The iron exchanged zeolite was then collected as a residue by discarding the supernatant. The collected modified zeolite was carefully washed with HCl solution (pH of 2.0) to remove occluded materials and surface-adherent salts and then washed with distilled water until it is chloride-free. A loosely ground mixture of 50 mg of air-dried exchanged zeolite and 50 mg of graphite powder was put in 5 mL tight Eppendorf tube to which 10 mg of polystyrene was added. 250 *μ*L of tetrahydrofuran and 350 *μ*L of dichloromethane were added to the tube, hand-shaken for 5 minutes, and further sonicated for three minutes. Finally, FeZ-G/GCE was prepared by casting 10 *μ*L of the suspension on the surface of a well-polished GCE and dried in air for about 30 minutes. The modified electrode was then activated by scanning it in a 0.5 M KCL in pH 7 PBS between -0.3 and 1.1 V until well-shaped characteristic peaks for Fe3+/Fe2+ appeared. The activated electrode is then prepared for use after scanning it in the buffer until stable wave is created.

## 3. Results and Discussion

### 3.1. Characterization of Iron (III) Doped Zeolite/Graphite Composite Modified GCE

#### 3.1.1. Electrochemical Impedance Spectroscopic Characterization

**Figure 1:**
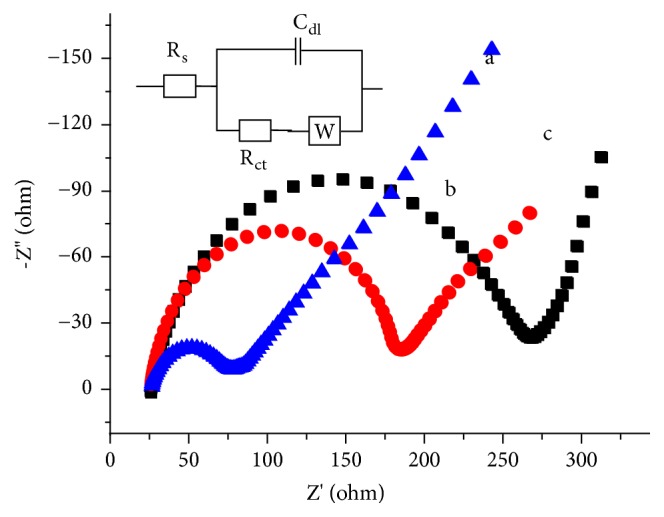
EIS Nyquist plot of (a) UGCE, (b) GGCE, and (c) FeZ-G/GCE in 0.1 M PBS (pH 7) containing 10 mM Fe(CN)_6_^3−^/^4−^ and 0.1 M KCl supporting electrolyte. Frequency range: 1.0-100,000 Hz; applied potential: +0.23 V; amplitude: 0.01 V. Inset: proposed equivalent circuit for the three studied electrodes.

The Nyquist plots for the investigated working electrodes under similar conditions are presented in Figure 1. As can be seen from the figure, all electrodes possess semicircles of different diameters associated with lines at 45 degrees. While the lines at 45 degrees depict diffusion of the electroactive species, the different semicircle diameters represent different charge transfer resistance and double layer capacitance. A semicircle with wider diameter for the composite modified electrode (curve c of Figure 1) than the graphite modified (curve b) and the unmodified glassy carbon electrode (curve a) indicated modification of the electrode surface with a material whose resistance to charge transfer is higher. An equivalent circuit (Inset of Figure 1) with charge transfer resistance (R_ct_) and Warburg constant (W) in series was proposed for the three electrodes. The result showed that the electrodes are different confirming the composite modification of the electrode.

#### 3.1.2. Cyclic Voltammetric Characterization

**Figure 2:**
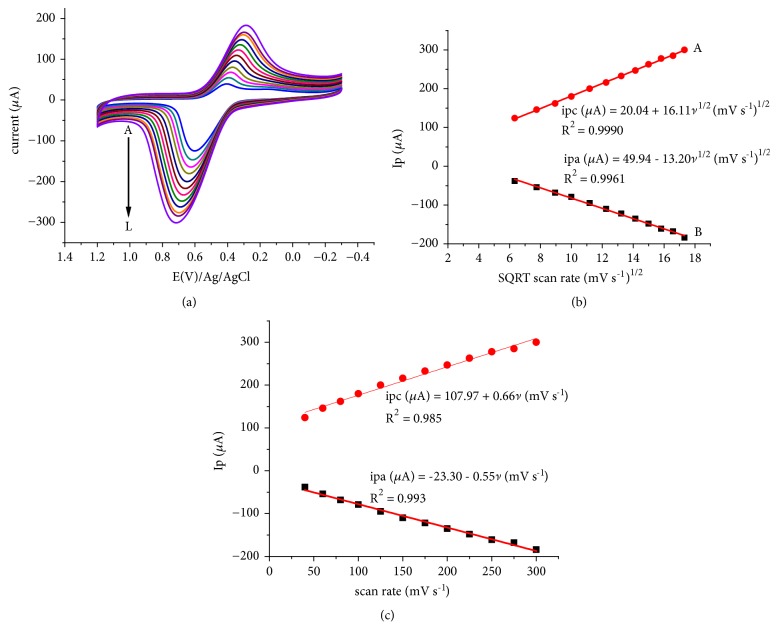
Cyclic voltammograms of stabilized FeZ-G/GCE in 0.5 mol L^−1^ KNO_3_ (in pH 7 PBS) at different scan rates (A-L: 40, 60, 80, 100, 125, 150, 175, 200, 225, 250, 275, and 300 mV s^−1^, respectively) (a); plot of peak current (Ip)* vs* square root of scan rate (b) and scan rate (c).

Figure 2(a) depicts the cyclic voltammograms of the composite modified glassy carbon electrode at various scan rates. Appearance of a pair of peaks in the potential range 0.2 to 0.8 V at all scan rates confirmed the incorporation of Fe^3+^ into the cavities of zeolite. Comparable correlation coefficients for the linear dependence of the peak current on the square root of scan rate (Figure 2(b)) and scan rate (Figure 2(c)) showed that the redox reactions were influenced by both diffusion of the electroactive species (Fe^2+^/Fe^3+^) from distant to the surface of the electrode where electron exchange is taking place and adsorption of the same species just at the surface. While the diffusion phenomena could be ascribed to the diffusion of the Fe^3+^ species from the cavities of zeolite to the surface and of the formed Fe^2+^ back to the cavities, the adsorption phenomena could be explained as the back and forth mass transfer of the electroactive species just from the surface [21, 23].

### 3.2. Electrochemical Behavior of Equimolar Mixture of UA and AA at Different Electrodes

**Figure 3:**
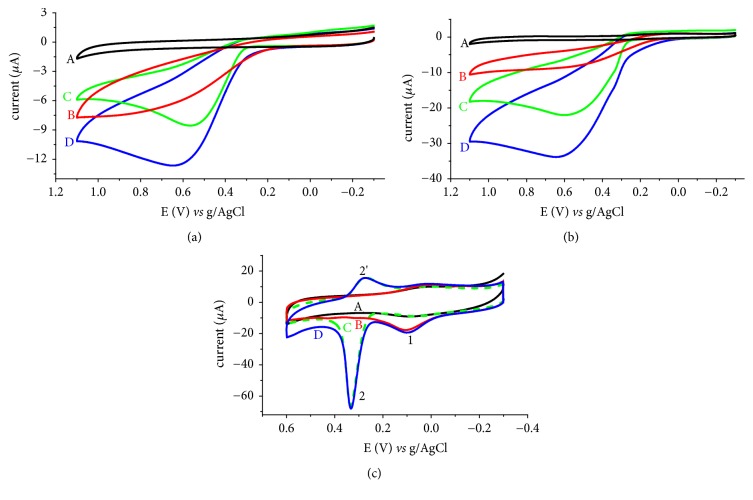
Cyclic voltammograms of (a) UGCE, (b) GGCE, and (c) FeZ-G/GCE in pH 7.0 PBS containing (A) no analyte, (B) 1 mM AA, (C) 1 mM UA, and (D) mixture of 1 mM AA and 1 mM UA at a scan rate of 100 mV s^−1^.

To evaluate the selectivity and catalytic property of the composite modified electrode for UA relative to the graphite modified and unmodified glassy carbon electrodes, cyclic voltammograms of each electrode were recorded for blank, UA, AA, and equimolar mixture of UA and AA (Figure 3).

Although relative peak current enhancement was observed at the GGCE for both the UA and AA, both electrodes were unable to detect UA and AA, separately (Figures 3(a) and 3(b)) which of course is in agreement with previously reported works [24]. On the contrary, the FeZ-G/GCE revealed well-resolved (separated) oxidative peaks with better current enhancement and overpotential reduction for AA and UA. Surprisingly, a reductive peak for UA was observed (peak 2′ of Figure 3(c)) at the FeZ-G/GCE which is absent not only at the UGCE and GGCE we studied but also at other reported modified electrodes. This observed electrocatalytic activity of the composite modifier may be attributed to the improved electron exchange and increased surface area. The electrocatalytic property of the composite modified electrode demonstrated by peak current enhancement, lowering of overpotential, separate peaks for AA and UA, and rise of an oxidative peak for UA confirmed superior candidacy of the iron doped zeolite/graphite composite modified glassy carbon electrode for determination of UA in real samples even in the presence of AA.

### 3.3. Effect of Scan Rate

**Figure 4:**
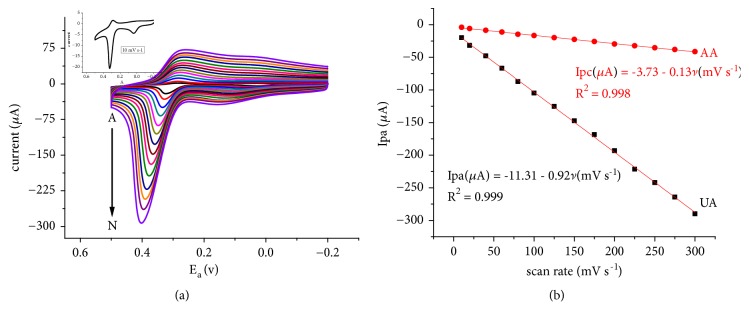
Cyclic voltammograms of a mixture of 1 mM UA and 1 mM AA pH 7.0 PBS at different scan rates (A-N: 10, 20, 40, 60, 80, 100, 125, 150, 175, 200, 225, 250, 275, and 300 mV/s, respectively) (a) and plot of peak current response for AA and UA against scan rate (b). Separated cyclic voltammogram of the same at scan rate of 10 mV s^−1^(Inset of (a)).

Sharp and intensive oxidative peaks for UA and weak peaks for AA with peak potential shift in the positive direction with scan rate (Figure 4(a)) confirmed the irreversibility of the oxidation reaction of UA at the surface of the modified electrode.

**Figure 5:**
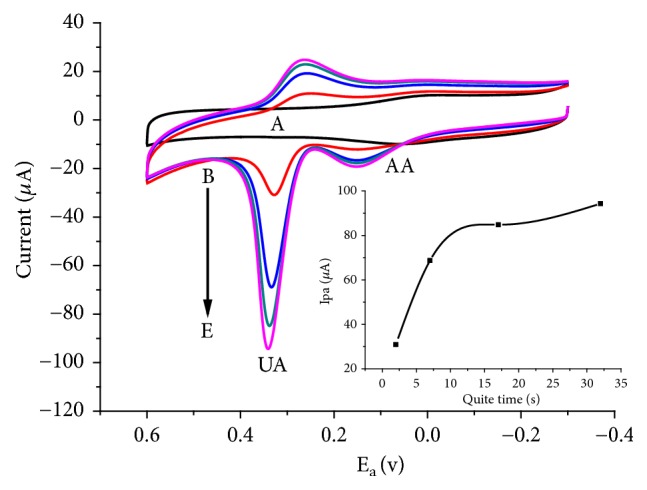
Cyclic voltammograms of FeZ-G/GCE pH 7 PBS containing (A) no UA and AA at a quite time of 2 s (A) and (B-E) containing 1 mM of UA and 1 mM of AA at various quite times (B-E: 2, 7, 17, and 32 s, respectively).

As can be seen from Figure 4(b), the peak current for both analytes is linearly related more with the scan rate than the square root of scan rate confirming the dominance of adsorption mass transport over diffusion. To further confirm the adsorption controlled oxidation reaction of UA, cyclic voltammograms of 1 mM UA in the presence of 1 mM AA were monitored at quite times 2 to 32 s (Figure 5). The oxidative peak current of UA at quite time of 7 s was found to be over 100% of the current at quite time of 2 s (Inset of Figure 5). Although the increment is lowered, still oxidative current increased with increasing quite time (time of exposure of the electrode surface to the analyte solution) confirming the adsorption controlled mechanism proposed above.

### 3.4. Effect of pH

**Figure 6:**
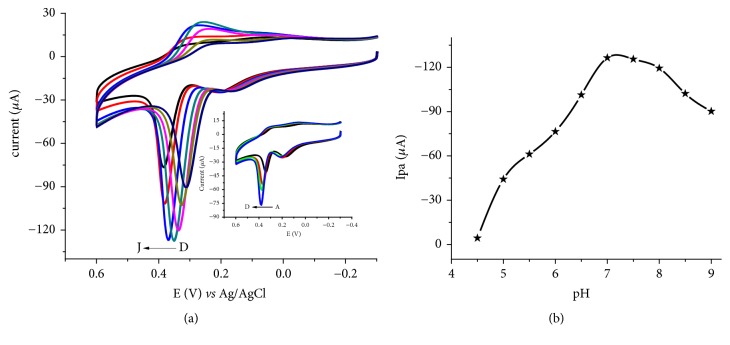
(a) Cyclic voltammograms of PBS of various pHs (D-J: 6.0, 6.5, 7.0, 7.5, 8.0, 8.5, and 9.0, respectively) containing 1 mM UA in the presence of 1 mM AA. Inset: voltammograms of 1 mM UA and 1 mM AA in PBS of pHs (A-D: 4.5, 5.0, 5.5, and 6.0, respectively). (b) Plot of peak current of UA against pH in the range 4.5-9.0.

To evaluate the effect of pH on the peak current and peak potential, cyclic voltammograms of the modified electrode in PBS in the pH range of 4.5 to 9.0 containing a mixture of 1 mM UA and 1 mM of AA were recorded (Figure 6(a)).

Whereas the oxidative peak potential of UA shifted in the positive potential direction leading to increased overpotential when pH is varied from 4.5 to 6.0 (Inset of Figure 6(a)), potential shift in the reverse direction and hence decreasing overpotential is noticed with increasing pH values from 6.0 to 9.0. This trend could still be ascribed to the type and magnitude of charges the surface of the electrode and analyte develop with pH. This observed peak potential shift with pH variation confirmed the participation of protons during the oxidation of UA at the surface of the composite modified electrode.

The peak current increased with pH from pH 4.5 to 7.0 and then started to decline at values higher than 7.0 (Figure 6(b)) demonstrating the participation of protons during oxidation of UA at the electrode surface. This trend could be ascribed to the electrostatic repulsion exerted between the positive charged zeolite due to the incorporated Fe^3+/2+^ and protonated UA (pKa = 5.4) whose strength decreases with increasing pH and the deprotonated zeolite and UA at higher pH values. Thus, pH 7.0 was taken as the optimum pH value where maximum current for uric acid can be recorded at the surface of the FeZ-G/GCE.

### 3.5. Determination of UA Content in Human Urine Sample

**Figure 7:**
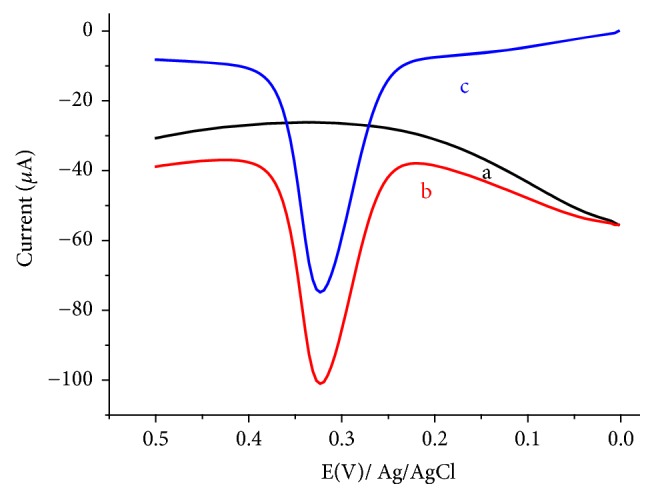
Square wave voltammograms of FeZ-G/GCE in pH 7 PBS containing (a) no UA, (b) 1 mM UA, and (c) corrected for blank (curve b and curve a).

Due to its sensitivity and ability to discriminate Faradaic from non-Faradaic current, square wave voltammetry was selected for the determination of UA in human urine. As depicted from Figure 7, a well-shaped voltammogram with an intense corrected oxidative peak current response at default parameters confirmed the applicability of SWV for the analysis of uric acid.

#### 3.5.1. Influence of Accumulation Potential and Time

**Figure 8:**
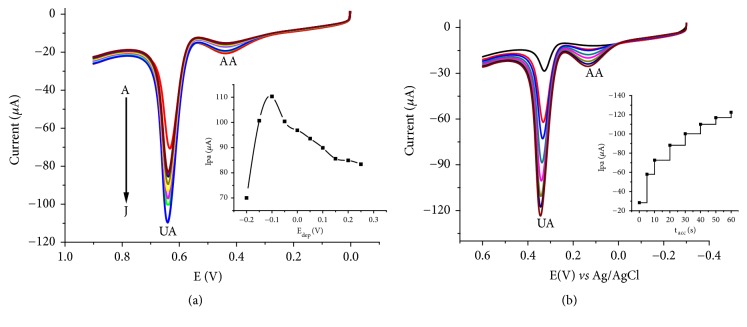
Square wave voltammograms of a mixture of 1 mM UA and 1 mM AA in pH 7 PBS at (a) E_acc_ of -200 to +250 mV and t_acc_ of 10 s, (b) E_acc_ of -100 mV and t_acc_ of 0 to 80 s, plot of peak current* versus *E_acc_ (Inset (a)), and plot of peak current* versus *t_acc_ (Inset (b)).

The observed dominance of adsorption over diffusion necessitated investigation of the effect of the adsorption potential and adsorption time on the oxidative peak current of UA. Square wave voltammograms of pH 7 PBS containing a mixture of 1 mM UA and 1 mM AA at deposition potentials in the range -200 mV and +250 mV and deposition time of 10 s are presented in Figure 8(a). The maximum peak current was recorded at an accumulation potential (E_acc_) of -100 mV (Inset of Figure 8(a)) and hence was taken as the optimum deposition potential.

Similarly, the peak current increased with increasing deposition time (Inset of Figure 8(b)) through the entire range of studied time. As a compromise between the enhanced peak current and accompanied analysis time, deposition time of 30 s where the slope of change has declined (Inset of Figure 8(b)) was selected as the optimum deposition time for further experiment.

#### 3.5.2. Analytical Performance of the Method under the Optimized Conditions

**Figure 9:**
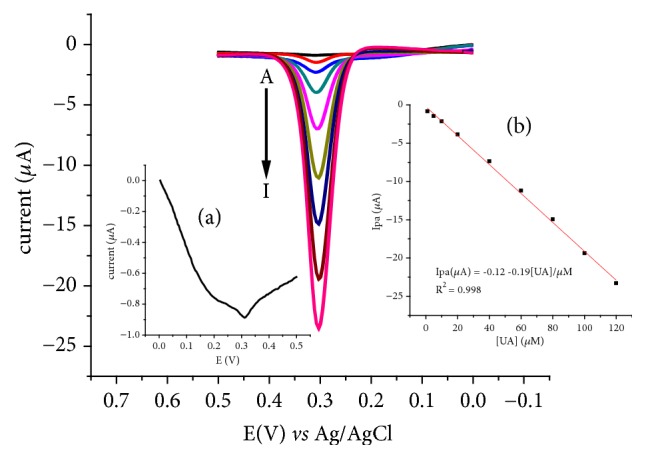
AdsASSWVs of FeZ-G/GCE in pH 7 PBS of various concentrations of UA (A-I: 1, 5, 10, 20, 40, 60, 80, 100, and 120 *μ*M, respectively). Inset: (a) amplified voltammogram for 1 *μ*M UA and (b) plot of oxidative peak current of UA against concentration of UA. Experimental conditions: E_acc_ = -100 mV; t_acc_ = 30 s; frequency = 15 Hz; amplitude = 25 mV; and potential step = 4 mV.

Adsorptive anodic stripping square wave voltammograms (AdsASSWVs) of FeZ-G/GCE in pH 7 PBS containing various concentrations of UA are presented in Figure 9. The oxidative peak current showed linear dependence on the concentration of UA in the range 1.0 to 120 *μ*M with determination coefficient (R^2^) and LoD (3s/m for n = 6) of 0.9976 (Inset of Figure 9) and 0.06 *μ*M, respectively.

#### 3.5.3. Analytical Applications

**Figure 10:**
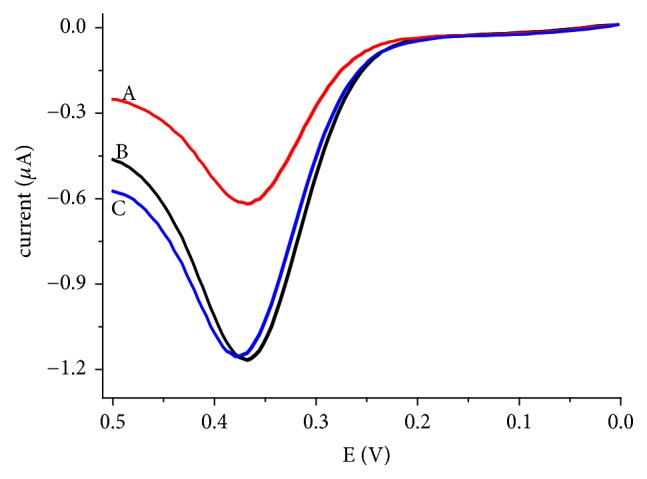
Selected AdsASSWVs of FeZ-G/GCE in 50 mL of pH 7 PBS containing 1 mL of centrifuged human urine sample collected from volunteers A, B, and C, respectively.

Iron doped zeolite/graphite composite modified GCE (FeZ-G/GCE) was applied for the direct determination of uric acid in urine samples collected from three young volunteers. Although triplicate voltammograms were recorded for each analyzed urine sample, one selected voltammogram for each urine sample is presented in Figure 10. The mean UA content of the urine samples calculated in mg per 100 mL of human urine is thus summarized in Table 1. As can be seen from the table, while the UA content of the samples from volunteers “B” and “C” is in the range for a healthy male (3.4 to 7.0 mg UA/100 mL urine sample), the UA content in the urine collected from the third volunteer “A” which was originally transparent relative to the others is below the range.

#### 3.5.4. Validation of the Developed Method

The developed method was validated using the precision between replicate measurements, recovery results for spiked UA in human urine sample, and selectivity of the method for UA in the presence of AA.

**Figure 11:**
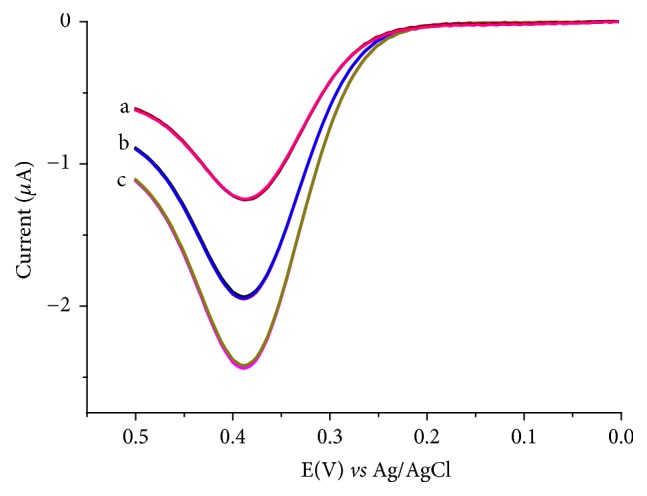
Representative AdsASSWVs of FeZ-G/GCE in pH 7.0 PBS containing (a) unspiked human urine sample, (b) human urine sample spiked with 3.5 *μ*M standard UA, and (c) human urine sample spiked with 7.0 *μ*M standard UA.

1 mL of centrifuged human urine (volunteer A) was added to each of three 50 mL flasks. While one flask was filled up to the mark with pH 7 PBS, the remaining were spiked one with 3.5 and the other with 7.0 *μ*M standard UA and finally triplicate voltammograms were recorded for each solution.  Figure 11 presents representative voltammogram for the unspiked (curve a) and spiked (curves b and c) urine solutions, whereas the recovery results are summarized in Table 2. Two important observations from the present study are excellent recovery results in the range 96.61 to 103.45% and very low RSD values (0.002 to 0.005) showing the validity of the method for determination of UA in unfiltered human urine with a complex matrix.

**Figure 12:**
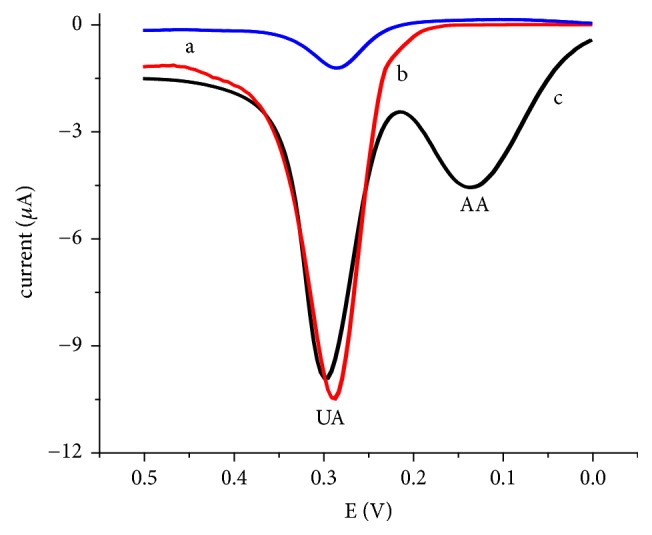
AdsASSWVs of FeZ-G/GCE in pH 7.0 PBS containing human urine solution (a) unspiked, (b) spiked with 50 *μ*M UA, and (c) b + 50 *μ*M AA.

To further validate the method, the selectivity of the composite modified electrode for UA was checked.  Figure 12 presents voltammograms of the composite modified electrode in both unspiked and spiked human urine samples. As can be observed from the voltammograms, the presence of AA caused the signal of the UA spiked human urine to decrease only by 4.2% confirming the selectivity of the method for UA and hence validating its applicability.

## 4. Conclusion

The developed iron (III) impregnated zeolite-graphite powder composite modified glassy carbon electrode (FeZ-G/GCE) was characterized using electrochemical impedance spectroscopy and cyclic voltammetry. Investigation of the electrochemical behavior of UA at the surface of the developed composite modified electrode revealed an irreversible oxidation at a reduced overpotential compared to at bare glassy carbon electrode indicating the catalytic property of the modifier towards oxidation of UA. Contrary to the electrochemical behavior of uric acid at the previously reported works, uric acid exhibited a reduction peak at the present electrode. The method using composite modified electrode was validated for its applicability for determination of UA in human urine samples. The oxidative current of UA at the surface of the electrode showed linear dependence on concentration in a reasonably wide range of concentration with a very good correlation coefficient value. The uric acid content of human urine samples collected from three male volunteers was found to be about the recommended level for healthy males. Excellent recovery and low interference results and normal uric acid content of the assessed urine samples added to the wide linear dynamic range validated the method for determination of uric acid in complex matrix systems like urine.

## Figures and Tables

**Table 1:** Summary of uric acid content in human urine samples collected from three volunteers.

Urine sample	Current*∗* (*μ*A)	Current ($\bar{X}\pm RSD$)/ (*μ*A)	Mean UA concentration in human urine (*μ*M)	UA content in mg/100 human urine sample
				($\bar{X}\pm RSD$)
A	0.615	0.625 ± 0.014	132.9	2.234 ± 0.012
	0.628			
	0.631			

B	1.165	1.179 ± 0.010	278.7	4.685 ± 0.009
	1.184			
	1.187			

C	1.175	1.183 ± 0.006	279.75	4.703 ± 006
	1.184			
	1.190			

*∗*triplicate measurement.

**Table 2:** Summary of the recovery analysis of 3.5 and 7.0 *μ*M UA in human urine (1:49 volume ratio of centrifuged urine to pH 7 PBS).

UA in unspiked urine solution (mg/50 mL)^*∗*^	Added	Found (mg UA/50 mL urine solution)^*∗∗∗*^	Percent recovery (%)
	(mg UA/50 mL)^*∗∗*^		
0.060 ± 0.002	0.029	0.090 ± 0.003	103.45%
	0.059	0.113 ± 0.005	96.61%

*∗*curve a; *∗∗*curve b; & *∗∗∗*curve c.

## Data Availability

All the generated cyclic and square wave voltammetric data used in this study (Electrochemical characterization of Iron (III) doped zeolite-graphite composite modified glassy carbon electrode and its application for AdsASSWV determination of uric acid in human urine) have been fully interpreted and are included within the article. Thus, anybody who wishes to use the data can use it following the publisher guideline.
